# Effect of Oral Physiology Parameters on In-Mouth Aroma Compound Release Using Lipoprotein Matrices: An In Vitro Approach

**DOI:** 10.3390/foods8030106

**Published:** 2019-03-21

**Authors:** Amparo Tarrega, Claude Yven, Etienne Semon, Patrick Mielle, Christian Salles

**Affiliations:** 1 CSGA (Centre des Sciences du Goût et de l’Alimentation), AgroSup Dijon, CNRS, INRA, Université de Bourgogne Franche-Comté, F-21000 Dijon, France; atarrega@iata.csic.es (A.T.); Claude.YVEN@agencerecherche.fr (C.Y.); etienne.semon@orange.fr (E.S.); patrick.mielle.inra@free.fr (P.M.); 2 ChemoSens Platform, CSGA, F-21000 Dijon, France

**Keywords:** lipoprotein matrix, chewing simulator, aroma compound, in vitro, oral parameters, flavor release

## Abstract

Temporal aroma compound release during eating is a function of the physicochemical properties of the food matrix, aroma compounds, and oral physiology of individuals. However, the influence of each parameter on the release of each aroma component should be clarified. Two flavored lipoprotein matrices varying in composition were chewed in a chewing simulator that reproduced most of the physiological functions of the mouth. Aroma compound releases (butanoic acid, 2-heptanone, ethyl butyrate, 3-octanone, and 2-nonanone) were followed in real time by direct connection of the device to APCI-MS (atmospheric pressure chemical ionization mass spectrometry). Each oral parameter was controlled and decoupled using the in vitro device. The food matrix composition had only a low impact on aroma compound release, but the controlled oral parameters had significantly different influences on the release of aroma compounds according to their physicochemical characteristics. The release of certain compounds seemed more sensitive to bite force, while others seemed more sensitive to the shearing angle. The salivary flow rate primarily influenced the more hydrophobic compounds. Significant interactions were also observed between shear angle, salivary flow rate, and lipoprotein matrix composition, mainly for the release of the more hydrophobic volatile compounds; this needs further investigations to be clarified.

## 1. Introduction

Food oral processing is a complex phenomenon involving several physical, chemical, and physiological mouth functions, leading to food breakdown and changes in in-mouth flavor release and perception before swallowing [1]. The main consequences of the oral process are the food matrix breakdown by mastication and impregnation of food by saliva, which are subjected to several physical, chemical, and biochemical operations [1,2]. During this step of food oral processing, the perception of flavor and texture are progressively affected. The release of aroma and taste compounds from food and their delivery to receptors are key factors leading to flavor perception. The breakdown of food into particles under eating conditions increases the surface area of the food that is exposed to saliva and air; this process drives the release of volatile compounds into the gas phase of the oro-nasal cavity. Moreover, aroma compound release is influenced by several physicochemical factors. During chewing and swallowing, aroma compound release, currently evaluated by nosespace analysis using online mass spectrometry [3], is dependent on food composition and texture, saliva and mastication parameters, as well as the nature of the aroma compounds in terms of partition coefficient and lipophilia [4,5,6]. In fat-containing foods, the retention and release of aroma compounds mainly depends on their solubility in the aqueous and oil phases and thus on their hydrophobicity [7,8], which is expressed by the log P value [9]. Most of the aroma compounds are more soluble in fat than in water. They are considered as hydrophobic (log P > 1) and less released in the gas phase from oil than from water, while the hydrophilic aroma compounds (log P < 1) are less retained by the oil phase and thus present quite similar air/water and air/oil partition coefficients. Consequently, the release of aroma compounds in the mouth from low-fat foods is higher than that with regular fat foods [6,10,11]. Flavor release and perception are mainly consequences of oral processing. In this process, saliva has various effects on aroma compounds, such as dilution, molecular interactions with saliva components, and enzymatic conversion. It also participates in aroma release. Consequently, it is a major actor in the perception of aroma [12]. A large inter-individual variability has been reported for salivary composition, in particular for alpha-amylase [13,14] and mucins [15]. The variability in composition may be related to variability in perception [16,17,18].

During this process, aroma compound release varies according to oral parameters [19] that are very dependent on individual oral physiology [5,20,21]. The release of flavor compounds from the food and their delivery to receptors are key factors leading to flavor perception. Temporal in vivo studies currently enable the direct release-perception tests because it is the most natural and realistic way to study flavor release and perception and to take into account all the complexity of the oral process involved in flavor release. However, such in vivo analyses may encounter limitations, such as major inter-individual differences due mainly to physiological and anatomical differences and moderate intra-individual reproducibility. In addition, the food sample must be acceptable to panelists, and only a limited number of samples can be evaluated daily. Another major limitation is the complexity of the oral functions and their interactions. The main consequence is difficulty for the specific role of each oral and product parameter to fully explain flavor release and perception. Thus, artificial devices have been developed to mimic human flavor release during food consumption under controlled conditions and to overcome these in vivo limitations [22,23,24]. It is a very hard task to simulate and integrate all the oral functions in a single and universal system. Thus, specificities and functionalities of each system should be conceived and tailored to address the asked scientific questions.

Several devices have been developed to simulate human flavor release and bolus formation under mouth-like conditions [23]. Many of them are limited to a controlled stirring or shearing of food particles in a liquid, while volatile compound released in the headspace is sampled [25]. More recently, devices were developed with higher functionality levels. Although they are far from mimicking all the main human mouth functions, mechanical food breakdown was more realistically reproduced, leading to relevant flavor release kinetics produced under controlled conditions. As examples, a mechanical chewing device was specifically developed to study the dynamic release of volatile compounds by direct connection to a proton transfer reaction mass spectrometer (PTR-MS) and sweeteners by saliva sampling analysis from chewing gums [26], although this system reproduced real human mastication patterns rather poorly. Additionally, an innovative and dynamic model mouth was specifically developed to investigate whether the intraoral pressure produced by the tongue affects the release of odorant compounds [27] by connecting online to a PTR-MS apparatus.

A chewing simulator allowing fluid sampling that integrates most of the main human oral functions has been described previously [24,28]. In particular, the shear force, compression force, salivary flow rate, and tongue function reflect average realistic physiological parameters obtained from human subjects and can be decoupled. In this study, we aimed to explore the variations in in vitro aroma release on model cheeses when oral parameters were decoupled using this chewing simulator [24]. The main goals were to test the suitability of the chewing simulator to study differences in flavor release according to parameters based on human oral physiology and to tentatively elucidate the influence of each oral parameter on the volatile compound kinetics, the nature of these compounds, and the composition of the model cheese.

## 2. Materials and Methods

### 2.1. Materials

#### 2.1.1. Chemicals

The volatile compounds used—butanoic acid, 2,3-butanedione, 2-heptanone, 2-nonanone, 3-octanone, ethyl hexanoate, ethyl butanoate, dimethyldisulphide—were food grade (Aldrich, St Quentin Fallavier, France). Pure water was prepared using a Milli-Q system (Millipore, St Quentin en Yvelines, France). Anhydrous milk fat (MF-Cormans, Goe-Limbourg, Belgique), powder of milk proteins (dry matter > 950 g/kg; free of fat) (Eurial Poitouraine, Nantes, France), sodium chloride (Jerafrance, Jeufosse, France), and rennet (Labo ABIA, Meursault, France) were used to prepare the lipoprotein matrices. All ingredients were food grade and assessed for microbiological safety.

#### 2.1.2. Lipoprotein Matrix

• Preparation

Flavored lipoprotein matrices (LPMs) were generated by the action of rennet on a mixture of milk protein, milk fat, NaCl, and water [29], to which the aroma compounds were added. Two LPMs were prepared, differing in their fat/milk powder ratio (Table 1): 0.5 for LPM1 and 1 for LPM2. The aroma compound solution was made of (in mg/kg): butanoic acid (10), 2,3-butanedione (3), 2-heptanone (5), 2-nonanone (5), 3-octanone (5), ethyl hexanoate (5), ethyl butanoate (4), and dimethyldisulphide (10) diluted in polyethyleneglycol (1 mL). Rennet extract containing 520 mg/l of chymosin (Sanofi Bio-Industries, Paris, France) diluted in 9 volumes of water was used for the coagulation step. This aroma formulation was optimized in the laboratory from internal cheese aroma analyses data to provide a standard cheese note that was acceptable by the consumers.

The LPM compositions are given in Table 1. The fat ratio was chosen based on a previous study [30] to be consistent with the fat content of standard marketed cheeses. Pure water, milk fat, milk protein powder, and NaCl were vigorously mixed with a Blender^®^ (Waring, Torrington, CT, USA) at medium speed (graduation 7) for 12 min. The mixture was cooled in a beaker, then placed in a thermostated bath at 32 °C. The pH was measured using a penetrometric electrode (Mettler-Toledo, Viroflay, France) and was adjusted at pH 6.5 using small aliquots of NaOH solution. After 30 min of resting, the aroma compound solution (0.5 mL) and rennet solution (4.8 mL) were added and mixed vigorously for 1 min. Prior to the coagulation, the mixture was immediately poured into a plastic bag and vacuum sealed, and then was completely immersed in a controlled-temperature bath at 32 °C for 3 h. The products were then stored at 4 °C until use.

• Instrumental texture analyses

LPM1 and LPM2 were analyzed by uniaxial compression at constant speed [31]. Cylinders of 3 cm length characterized by a ratio length/diameter of 1.3 were prepared for each LPM using a die-cutter of 2.3 cm internal diameter. Each sample was placed in a hermetically closed plastic cup at 19 °C for 15 min before experiments. The resistance strength, developed as a function of the deformation of the sample, was measured at 15 °C using a texture analyzer (TA-XT2, Stable Micro Systems Ltd., Champlan, France). The displacement speed of the superior plate (10 cm diameter) was 0.8 mm/s, and the samples were compressed until a deformation rate of 80% of their initial height.

### 2.2. In Vivo Mastication

Relevant physiological parameters (salivary flow rate, number of chew, shear angle, total duration of mastication, time between two chews, chew rate) were measured on three human subjects (volunteer staff of the laboratory; males; between 25 and 45 years old) without any pathology to obtain a range of representative and realistic values to use as inputs into the chewing simulator (Table 2). The procedure to analyze aroma release was the same as described in [32]. In particular, they were asked to chew the LPM cubes in their own way without any specific instruction. They were informed of the experiment objective and conditions. They gave their informed consent before they participated in the study. The study was conducted in accordance with the Declaration of Helsinki, and the protocol was approved by the relevant institutional and national regulations and legislation (Comité de Protection des Personnes Est-1, N° 2013/64–IDRCB 2013–A01084-41 on 21 November 2013).

#### 2.2.1. Salivary Flow Rate

The whole saliva flow rate was measured under real conditions of model cheese eating with LPM2, as it was reported in a previous study that salivary flow rate was not found to vary for the same subject when eating different model cheeses samples [5]. The total flow rate was determined by calculating the weight difference. To determine the salivation with both mechanical and chemical stimulations due to the presence of LPM in their mouths, the subjects were instructed to swallow just before introducing 5.0 ± 0.1 g of model cheese in their mouth and then to chew over a one minute period without swallowing. Next, the subjects spat out all the bolus and saliva in a tare flask, quickly cleansed their mouths with 10 g of mineral water, and spat in the same tare flask. All the measurements were performed in triplicate and at the same time of day for all subjects.

#### 2.2.2. Electromyography Recording

The electromyography (EMG) recordings were conducted according to Mioche et al. [33]. The left and right superficial masseters and anterior temporalis muscles of each volunteer were located by palpation when the teeth were clenched. After careful cleaning of the overlying skin, two surface electrodes (Bionic France, Ternay, France) coated with a conductive paste were fixed 2 cm apart lengthwise along each muscle with an adhesive. An additional earth electrode was attached to the subjects’ ear lobes. Each subject was instructed to naturally eat 5 g of model cheese. The experiments were performed in triplicate for each product.

After signal rectification, several variables were analyzed for the complete sequence of mastication, starting at the moment of food intake and ending at the last swallow—chewing time (total sequence duration before the last swallow), number of chews during the chewing time, chewing rate per minute, the mean voltage of each burst, the sum of the integrated areas of all individual bursts in the sequence (burst duration multiplied by its mean voltage expressed in mV·s), previously called muscle work, and the mean work (total work divided by the number of bursts).

#### 2.2.3. Motion Capture

An infrared light-emitting diode (LED) was attached to the chin to follow the movements of the lower jaw, a second reference LED was attached to the brow, and a third one was attached to the laryngeal prominence to follow swallowing events. The LEDs were attached using a double-sided adhesive strap. The positions of the LEDs were recorded using an optical motion capture system (Northern Digital Optotrak, Radolfzell, Germany). The recording of jaw movement during chewing was superimposed to correctly align with the EMG recording to better repair chewing events. It was also used to calculate the average shearing angle of the lower jaw used as a parameter for the chewing simulator.

All the data gathered using these three methods were used as input parameters in the chewing simulator methods.

### 2.3. In Vitro Mastication

#### 2.3.1. Chewing Simulator

A chewing simulator specially developed for in vitro flavor release study was used [24,28]. Particularly, it was fitted with a special valve for the online sampling of the gas phase. The chewing simulator was connected to an APCI-MS (mass spectrometer equipped with an atmospheric pressure ionization source) apparatus (Esquire-LC ion trap, Bruker Daltonique, Wissembourg, France). The operating procedure for mass spectrometry was the same as described in [32].

**Figure 1:**
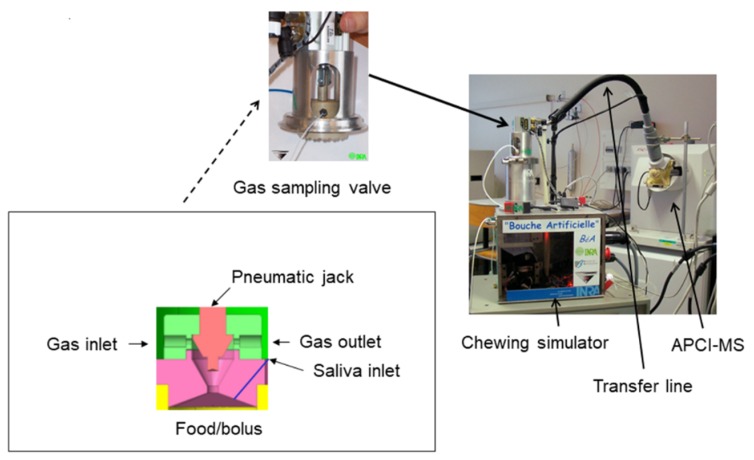
Chewing simulator connected to the atmospheric pressure chemical ionization mass spectrometer (APCI-MS) for online and real time analyses of aroma compound release under in vitro mastication conditions with details of the gas sampling valve.

The upper part of the chewing simulator (Figure 1) was made of the upper jaw and conical palate containing the saliva inlet injector located approximately 10 mm from the upper teeth crown and central gas valve. The sampling valve was in close position when the distance between the tongue and palate was lower than 10 mm to avoid the valve and tubing from becoming contaminated and clogged. A bypass allowed the gas to flow towards the analyzer to maintain the baseline when the sampling valve was off. When the valve was open, volatiles in the headspace of the chewing simulator passed through the valve and were transferred to the mass spectrometer via the Venturi effect regulating the gas flow rate. On the opposite side of the valve, a vent allowed external air to enter the cell. All the surfaces of the chewing simulator in contact with the food were made of polyetheretherketone (PEEK). The transfer line was a deactivated fused silica capillary tube [24,28].

The bite force (BF; between 20 and 35 daN), salivary flow rate (between 1 and 4 mL/min), and shearing angle (SA; 3 and 5°, respectively, corresponding to a 1/8 and 1/5 tooth shift, respectively) were the variable parameters. Three replicates were performed for each measurement condition and for each of the two LPMs (Table 1). Artificial saliva containing minerals and mucin was used [34]. A syringe pump controlled by the chewing simulator software ensured the constant artificial salivary flow rate.

#### 2.3.2. Volatile Compound Release Measurements

**Figure 2:**
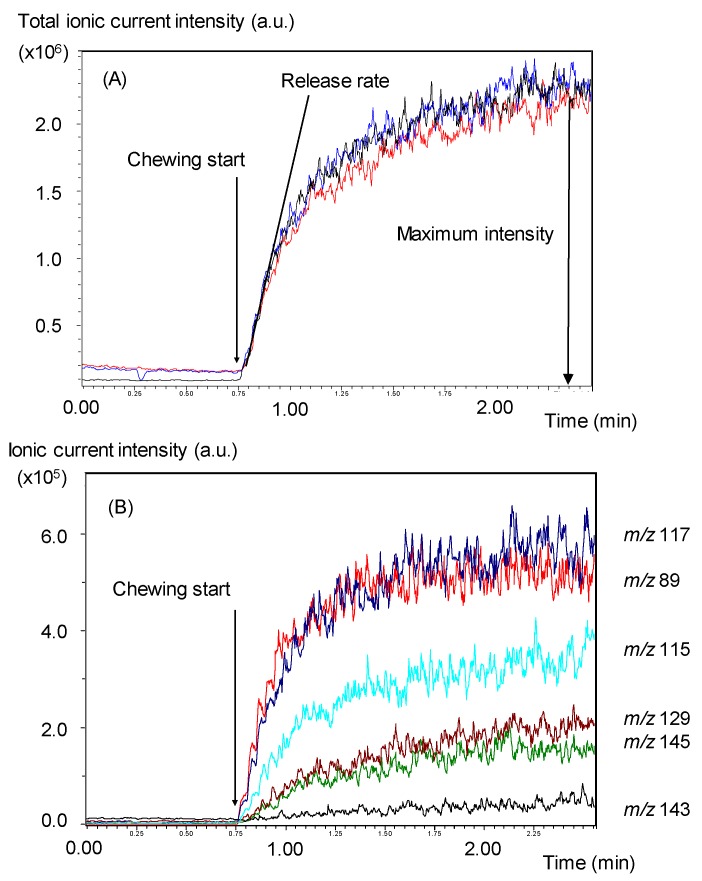
Example of in vitro temporal aroma compound release curves obtained by online coupling of the chewing simulator with the APCI-MS apparatus (3 replicates). (**A**) The total ionic current presented in ordinate is representative of the overall volatile compounds concentration in the gas phase according to the time. (**B**) Ionic current intensity for each detected ion in function of the chewing time. Conditions: initial volume of saliva: 1 mL; salivary flow rate: 1 mL/min; compression force: 25 DaN; shearing angle: 3°. Volatile compounds in lipoprotein matrices (µL/kg): butanoic acid (10); 2,3-butanedione (3); 2-heptanone (5); 2-nonanone (5); 3-octanone (5); ethyl hexanoate (5); ethyl butanoate (4); dimethyldisulphide (10). Ions: *m/z* 117 (ethylbutanoate); *m/z* 89 (butanoic acid); *m/z* 115 (2-heptanone); *m/z* 129 (3-octanone); *m/z* 145 (ethylhexanoate); *m/z* 143 (2-nonanone).

The nosespace sampled online from a subject or the chewing simulator was drawn at 30 or 80 mL/min and flowed through a heated capillary transfer line (150 °C, 0.53 mm i.d.) into an ion-trap mass spectrometer Esquire-LC (Brucker Daltronique, Wissembourg, France) equipped with a modified APCI source [35]. Aroma compounds were ionized by a 5-kV positive ion corona pin discharge. After checking the main ions for each volatile compound using single aroma compound solutions as references, the data were collected for detectable ions corresponding to protonated aroma compound molecules incorporated in the model cheeses (Table 3). The intensity of the signal was expressed in arbitrary units. In all cases, the room air and exhaled air by the subject or by the chewing simulator—before introducing the food sample in the mouth—was recorded and then was subtracted from the records obtained when chewing foods. Aroma release, jaw muscle contraction, and motion were recorded at the same time for the three subjects. Two parameters were extracted from each release curve—the release rate (RR) taken at the beginning of the chewing process, and magnitude, defined as the difference between the level of release before and after the chewing process (Cmax) (Figure 2).

The hydrophobicity of the aroma compounds (Log P) was calculated with BIOVIA discovery studio software (version 2017, BIOVIA Corporate Europe, Cambridge, UK) according to Ghose et al. [35].

### 2.4. Statistical Analyses

The data were processed using Stat SAS system release 6.12 (SAS Institute Inc, Cary, NC, USA). Analyses of Variance (ANOVA) were performed at the level α = 0.05 to study the variation in oral parameters (model including product, subject, and product * subject effects) and aroma compound release parameters (model including product, oral parameter, product * oral parameter). The means were compared using the Newman-Keuls multiple comparison tests.

## 3. Results

The two lipoprotein matrices used in this study were different only in their fat/protein ratio (0.5 and 1). These food matrices were comprised of milk fat and proteins that were coagulated using rennet. Their composition and rheological characteristics are given in Table 1. Among the four rheological parameters obtained by uniaxial compression, significant differences were observed for the modulus of deformability (MD). Thus, the lipoprotein matrix LPM2 with higher dry matter and fat content showed a higher MD value, indicating a harder structure than LPM1. The LPMs were aromatized with a very simple volatile compound composition to mimic a typical cheese flavor (Table 1). However, two of them, 2,3-butanedione and dimethyldisulphide, were not detectable by APCI-MS because of the low sensitivity for their detection. Thus, the presented data concern only the five detectable ions corresponding to butanoic acid, 2-heptanone, 2-nonanone, 3-octanone, ethyl hexanoate, and ethyl butanoate (Table 1).

### 3.1. In Vivo Aroma Compound Release

The oral physiological characteristics of the three subjects (named A, B, and C) were evaluated mainly to limit the decoupled oral functions of the chewing simulator to simulate mastication and salivation with realistic values. The obtained results with the three subjects were in agreement with previous works [5,20,21], thus we considered that the obtained results could be used as relevant information for the in vitro experiments. These in vivo results are briefly presented below.

The oral physiological parameters recorded on the three subjects are reported in Table 2. No influence of LPM or the interaction subject LPM was observed on chewing parameters. The influence of subjects was observed on most of the measured oral parameters. The salivary flow rate was evaluated for each panelist using a lipoprotein matrix (LPM2) to obtain representative values with the food product, considering mechanical and chemical stimulations specifically provided by the studied products. The salivary flow rates of A and B were significantly higher than that of C. By contrast, the shearing angles of A and B were significantly lower than that of C. The number of chewing cycles and chewing duration of B and C were similar but significantly higher than those of A. The maximal amplitude of chewing cycle and total muscle work were significantly different for each of the subjects in the decreasing order: B > C > A.

Concerning aroma compound release, no influence of the LPM on either the RR value or on Cmax—regardless of the ion considered—was observed. The overall effect of the subjects on RR was significant (F = 11.94, *p* = 0.00002) with the decreasing order: B > C > A.

The effects of the subject factors on Cmax and RR and interaction with LPM are reported in Table 3, confirming the large inter-individual variability. Correlations between the aroma release and chewing parameters were found. Cmax and the number of chewing cycles were well correlated regardless of the ion considered. For each ion, the significant correlations were as follows: butanoic acid (*m/z* 89; r = 0.48, *p* = 0.041), 2-heptanone (*m/z* 115; r = 0.68, *p* = 0.002), ethyl butyrate (*m/z* 117; r = 0.57, *p* = 0.012), 3-octanone (*m/z* 129; r = 0.55, *p* = 0.018), 2-nonanone (*m/z* 143; r = 0.58, *p* = 0.011), and ethyl hexanoate (*m/z* 145; r = 0.47, *p* = 0.044). Correlations between Cmax and total muscle work for ions 89 (r = 0.67, *p* = 0.002), 115 (r = 0.52, *p* = 0.027), 117 (r = 0.67, *p* = 0.002), and 143 (r = 0.578, *p* = 0.012) were also reported. Moreover, for ion 89, correlations between RR and total muscle work (r = 0.54, *p* = 0.021) and between RR and the salivary flow rate (r = −0.72, *p* = 0.026), were observed. However, because of the low number of observations, these in vivo data could not be further interpreted and should be further validated with a larger number of panelists. They were mainly used as a basis for our choice of in vitro chewing parameters.

### 3.2. In Vitro Chewing Process

The good superposition of the total ionic current obtained for three replicates made under the same conditions shows the good reproducibility of the in vitro aroma compound release measurements made by direct coupling between the chewing simulator and APCI-MS. For example, three replicates obtained with LPM1 are shown in Figure 2. The reproducibility was generally better than what can be observed in in vivo studies that are sensitive to inter- and intra-individual variability and reproducibility [36,37]. The pattern of the temporal curve obtained while using the device was different from the typical symmetrical curve, with a pre-swallowing and a post-swallowing phase that could usually be observed for in vivo temporal compound release kinetics [38]. On the in vitro curves, the absence of the post-swallowing phase was due to the absence of a swallowing function of the chewing simulator. Thus, only the release rate (RR) and maximum concentration of released compounds (Cmax) could be extracted from the pre-swallowing phase of the curve.

The release kinetics for each detectable ion and their analyses were followed according to their volatility and affinity for the different phases after extraction of the initial slope, indicating the release rate and maximum intensity from these curves. If each ion was considered independently, it was clear that differences in aroma compound release were observed according to the physicochemical characteristics of the volatile compound and the oral parameters. For each ion, all the obtained results are reported in Table 4. The maximum concentration values of less hydrophobic compounds (butanoic acid, 2-heptanone, and ethyl butyrate) were mainly affected by bite force and shear angle. A higher bite force and shear angle resulted in a higher aroma release. The aroma release parameters of more hydrophobic compounds (3-octanone, 2-nonanone, and ethyl hexanoate) were, in general, affected by the salivary flow rate, but the effect depended on the type of lipoprotein matrix and shear angle, as observed by the significant interaction effects.

## 4. Discussion

### 4.1. Texture of the Lipoprotein Matrices

The two LPMs mainly varied in their fat/milk protein ratio by a factor of two. This parameter is an important factor in aroma compound release and texture variability [4]. The rheological properties of the two LPMs presented in Table 1 only differed in their modulus of deformability, indicating a lower elasticity of LPM2 than that of LPM1. Although the LPMs required sufficient chewing activity to be broken in the mouth, their difference in elasticity did not affect the oral parameters of the panelists (Table 2). It was noticed that, with a larger variation in texture, differences in the oral parameters were reported [4,39,40], and during all the in vivo process, effects of bolus hardness on masticatory kinetics were observed [2,41,42]. Thus, the absence of a difference in the oral parameters between the two LPMs for each panelist could be explained by the difference in texture between the two LPMs being insufficient to trigger to a change in the mastication pattern of the subjects.

### 4.2. Effect of Bite and Shear Forces on In Vitro Aroma Compound Release

Concerning in vitro aroma compound release, mechanical mastication increased the release of the aroma compounds, as already mentioned by several works performed with in vitro systems [22,43,44].

In our study (Table 4), the less hydrophobic volatile compounds—butanoic acid, 2-heptanone, and ethyl butanoate—were more released when the bite force and shearing angle were higher (Table 4). This showed the importance of these two mechanical parameters for the release of these compounds. The progressive increase in the exchange area of the particles due to the breakdown of the matrix could easily explain this observation. A high bite force led to better food breakdown in the mouth. However, no effect on the release rate was found in either BF or SA, suggesting that these two parameters did not influence the kinetics of release of the less hydrophobic volatile compounds but only the overall quantity of the released components.

For the more hydrophobic compounds, the effects of BF and SA were less clear and seemed more specific to the nature of the volatile compound. Concerning the maximum released concentration, a significant effect of BF (but not of SA) was observed for 3-octanone, while a significant effect of SA (but not of BF) was observed for 2-nonanone. For ethyl hexanoate, only a significantly higher release rate was observed with an increase in the shearing angle. These volatile compound behavior differences were rather difficult to interpret and need further specific investigations to be more clearly understood. The data reported in Table 4 clearly show the impact of the shearing angle in the aroma compound release intensity. Generally, the overall mechanical effect of the jaw on food breakdown and aroma compound release is the result of shear and compression forces. In this study, shear force, related to the horizontal movement, could be independent of the bite force related to the vertical movement of the chewing functions reproduced in vitro, showing their own impact on aroma compound release. The closing angle and width of the chewing loop were found to be significantly correlated with the toughness and modulus of elasticity, which were two mechanical properties of the food, whereas the vertical movement was not correlated [45]. In addition, it was reported that, in vivo, the lateral chewing motion was dependent on the consistency of the food, although differences between subjects were observed due to the retro control by mechanoreceptors [46]. A greater consistency of foods resulted in a larger lateral chewing motion of the lower jaw. These observations were in favor of a high involvement of the shearing forces in the food breakdown of hard foods and bolus formation, leading to higher spreadability of the bolus and potentially higher emulsification of the bolus in saliva, thus increasing the exchange surface with air. This could influence aroma compound release differently according to the nature of the volatile compounds.

### 4.3. Effect of Salivary Flow Rate on In Vitro Aroma Compound Release

A significant effect of the salivary flow rate on the aroma compound release was observed only for the most hydrophobic compounds—ethyl hexanoate, 3-octanone, and 2-nonanone. For these compounds, an increase in the salivary flow rate led to a decrease in the maximum concentration of released compounds, and in addition, a decrease in the release rate for ethyl hexanoate, which was the least hydrophobic of these three molecules. We have to consider here that the food matrix is a complex medium. Consequently, the behavior of volatile compounds and their partition between the different phases cannot be explained only by their hydrophobicity, as is the case for simple models. Despite the hydrophobic character of these compounds, the progressive increase of artificial saliva volume should be in favor of their displacement to the water phase, leading to a higher release in the air phase because of their hydrophobicity [48]. Although the retention/release phenomenon is not understood completely in complex food matrices [49], a possible explanation is the effect of dilution by an increase of saliva volume, increasing the capacity of the water phase to retain more hydrophobic volatile compounds, leading to a partition less in favor of the gas phase. This effect does not affect the less hydrophobic molecules that are more soluble in water. It cannot be excluded that this effect could also be due to interactions with salivary proteins that are progressively introduced with saliva during the chewing process, but this is not generally described as the major phenomenon that explains volatile compound retention [12]. Previous in vitro studies have shown that increasing salivary flow rate decreased the aroma released from bell peppers [44], and the addition of saliva and water decreased the release of some aroma compounds from coffee brews [50].

However, the impact of the salivary flow rate on aroma compound release is not so clear and varies according to the studies and conditions. In an in vivo study, Pionnier et al. found no influence of the salivary flow rate on the release of aroma compounds of various polarities during the chewing of a model cheese [38]. In another in vivo study, it was reported that the salivary flow rate did not vary among cheese samples, but, at the end of mastication, the amount of saliva in boluses differed depending on the hardness and fat content of cheeses [5]. The authors suggested that the combined effects of both the chewing process and salivary flow rates were responsible for differences in aroma compound release between subjects. However, it was not possible to independently decouple the effects of each phenomenon.

### 4.4. Effect of Oral Functions and Food Composition Interactions on In Vitro Aroma Compound Release

Different interactions between oral parameters and composition factors were observed, thus modulating the main effects described above.

Although fewer effects were observed concerning the release rate, a significant interaction showed that, only for 2-heptanone, the release rate was significantly higher when the shearing angle and fat content were the highest, also corresponding to the hardest lipoprotein matrix, which contained less water and fewer proteins. This effect seemed rather complex and specific to this compound.

For the more hydrophobic volatile compounds, salivary flow rate effect showed significant interaction with the LPM and shear angle factors, indicating that the effect salivary flow rate had on aroma compound release depended on the food matrix and the extent of chewing. The effect of the salivary flow rate showed interaction with other factors. Significant interaction with the lipoprotein matrix was observed for the maximum concentration of the more hydrophobic aroma compound released—apart from 2-nonanone, for which the interaction was observed for RR. The maximum concentrations of the released 3-octanone and ethyl hexanoate were significantly higher at the high salivary flow rate when the fat content was highest, while it was the opposite for ethyl butanoate, which was the less hydrophobic volatile compound for which this interaction was significant. 2-nonanone was released significantly more slowly when the food matrix contained more proteins and less fat, and the salivary flow rate was low. Slight differences occurred between the effects of interactions between the volatile compounds. However, the resulting observed effect was that the combination of the low salivary flow rate and low fat and high protein content of the food matrix was in favor of a lower release of hydrophobic volatile compounds, while the combination of a high salivary flow rate and high fat and low protein food matrix was in favor of a higher release. These observations were consistent with previous observations [4,51]. However, this interaction was not easy to interpret because several composition parameters varied. This needs further in vitro experiments to be unraveled.

In in vivo studies, it is often questionable whether such effects of texture and composition of the food matrices on aroma compound release are due to physicochemical interactions between the food components and volatile compounds or due to a change in the chewing behavior because of the retro-control of the chewing function by texture perception [39,40]. Here, the control and decoupling of the chewing parameters using the device allowed us to discard the second assessment.

Significant interactions between the salivary flow rate and shearing angle were also observed for the release of the more hydrophobic volatile compounds. For 3-octanone and ethyl hexanoate, the maximum concentration of released compounds was significantly higher for the higher salivary flow rate and shearing angle than for the other cases due to a more efficient extraction with an important mixing effect in a larger quantity of solvent. A lower release rate of 2-nonanone and ethyl hexanoate was observed when low shearing and low salivary flow rate were combined. In this case, a lower mixing combined with a low salivary flow rate led to fewer exchanges between the gas phase and liquid phase and thus to a slower release.

## 5. Conclusions

The chewing simulator can precisely reproduce the compression and shear forces of human jaws, causing temporal food breakdown and compound release. In this in vitro study, each oral parameter and the interactions between them could differently influence temporal aroma compound release according to the nature of the aroma compounds. Moreover, we were able to control each parameter separately so that the effects could be measured one at a time in isolation, particularly for the bite and shear forces on aroma compound release.

In most in vivo studies dealing with relationships between oral processing and temporal aroma compound release, it is difficult to attribute the relative importance of each single oral parameter to the individual aroma compound release pattern. Moreover, individuals adapt their individual eating pattern to the texture and consistency of the food matrix, leading to changes in aroma compound release [5,52]. Thus, generally, the part of the in-mouth release of stimuli due to food characteristics and the adaptation of food oral processing according to texture perception are difficult to differentiate. The possibility of controlling and decoupling oral functions with our chewing simulator device could allow overcoming limitations of the in vivo aroma compound release studies.

Using this device, we were able to explain the temporal release of the studied volatile compounds by the effect of the components of the surrounding media and physical constraints due to mastication on aroma compound release. In particular, we reported effects of salivary flow rate varying according to the hydrophobicity of the volatile compounds and a clear effect of bite force and shear angle on the less hydrophobic volatile compounds, leading to a higher release. The disconnection between the bite force and the shearing angle allowed us to highlight the importance of the shear angle on flavor release during chewing. However, for the release of the more hydrophobic volatile compounds, the effects of the bite force and shear angle seemed more typical of the nature of the compounds and thus were more difficult to interpret. Significant interactions were also observed between shear angle, salivary flow rate, and lipoprotein matrix composition, mainly for the release of the more hydrophobic volatile compounds, which needs further investigation to be clarified.

Thus, this tool allows identification of the main physical or physiological phenomena explaining the active release, as well as validation of the proposed assumptions to model in silico aroma compound release. Additional functionalities that have been shown to be important for aroma compound release are lacking. Their integration in this device and the improvement of the system are under investigation.

## Figures and Tables

**Table 1:** Composition and characteristics of the lipoprotein matrix (LPM) samples (% of total mass).

	LPM 1	LPM 2
**Composition** for 500 g		
Water (g)	310 (62)	275 (55)
Anhydrous milk fat (g)	61.5 (12.3)	110 (22)
Milk powder (g)	123.5 (24.7)	110 (22)
NaCl (g)	5 (1)	5 (1)
Aromatic solution ^a^ (mL)	0.5 (0.1)	0.5 (0.1)
Rennet ^b^ (mL)	4.8 (0.96)	4.8 (0.96)
Fat/Milk Protein powder	0.5	1
**Rheological characteristics**		
MD (kPa)	32.78 *	44.38 *
Df (-)	0.42	0.40
Cf (kPa)	23.36	23.88
Wf (kJ/m^3^)	4.14	4.34

* Significantly different (F = 7.76; *p* = 0.022); ^a^ Composition: butanoic acid (10 µL), 2,3-butanedione (3 µL), 2-heptanone (5 µL), 2-nonanone (5 µL), 3-octanone (5 µL), ethyl hexanoate (5 µL), ethyl butanoate (4 µL), dimethyl disulphide (10 µL), and polyethyleneglycol up to 1 mL; ^b^ active chymosin 520 mg/L, dilution 1/10; MD: modulus of deformability, Df: fracture strain, Cf: fracture stress, Wf: fracture work.

**Table 2:** Physiological results obtained for each subject during chewing LPM1 and LPM2.

Subject	Product	Salivary Flow Rate (mL/s)	Chewing Duration (s)	Total Work of Muscle (mV s)	Number of Chewing Cycles	Mandible Force (daN)	Shearing Angle (°)
A	LPM1		13.5 ± 2.9	1.01 ± 0.31	25.0 ± 8.5	13.3 ± 1.1	2.6 ± 0.4
	LPM2	4.0 ± 0.4	15.1 ± 4.9	1.06 ± 0.13	21.3 ± 4.0	11.5 ± 0.5	2.7 ± 0.6
B	LPM1		23.1 ± 6.7	3.48 ± 0.25	33.7 ± 7.0	21.6 ± 1,5	5.0 ± 0.5
	LPM2	2.8 ± 0.2	24.9 ± 0.7	3.75 ± 0.23	38.0 ± 0	20.4 ± 0.5	4.7 ± 0.5
C	LPM1		28.0 ± 2.4	2.92 ± 0.19	39.0 ± 3.6	16.4 ± 0.4	3.1 ± 1.5
	LPM2	3.5 ± 0.2	23.7 ± 0.8	2.60 ± 0.16	33.7 ± 3.1	16.9 ± 0.8	3.0 ± 0.3

LPM1: lipoprotein matrix with 0.5 fat/milk protein ratio; LPM2: lipoprotein matrix with 1 fat/milk protein ratio; 3 replicates for each of the 3 subjects (A, B and C).

**Table 3:** In vivo aroma compound release. Fisher statistics from Analyses of Variance (ANOVA) of the temporal aroma compound release parameters. Only significant results (*p* < 0.05) are reported. Cmax: maximum released concentration; RR: release rate; LPM: lipoprotein matrix.

Ions (*m/z*)	Release Parameters	F	*p*	Subject Effect
89	Cmax	5.66	0.018	B > (C = A)
	RR	7.44	0.007	B > (C = A)
115	Cmax	7.71	0.007	(B = C) > A
	RR	9.36	<0.001	B > (C = A)
117	Cmax	5.47	0.021	B ≥ C ≥ A (B > A)
	RR	5.44	0.021	B ≥ A ≥ C (B > C)
129	Cmax	5.98	0.015	C ≥ B ≥ A (C > A)
	RR	7.98	0.006	(B = A) > C
143	Cmax	5.88	0.016	(B = C)> A
	RR	7.34	0.008	B ≥ A ≥ C (B > C)
	RR	9.31	0.003	Subject*LPM: B-LPM2 > B-LMP1
145	RR	4.58	0.033	B ≥ A ≥ C (B > C)

*m/z*: mass/charge ratio; *m/z* 89 (Butanoic acid); *m/z* 115 (2-Heptanone); *m/z* 117 (Ethyl butanoate); *m/z* 129 (3-Octanone); *m/z* 143 (2-Nonanone); *m/z* 145 (Ethyl hexanoate). F: Fisher value calculated by ANOVA; * indicates interaction between parameters.

**Table 4:** In vitro aroma compound release from lipoprotein matrices. Fisher statistics from Analyses of Variance (ANOVA) of the temporal aroma compound release parameters. Only significant results (*p* < 0.05) are reported. Cmax: maximum released concentration; RR: release rate; LPM: lipoprotein matrix; BF: bite force; SA: shearing angle; SF: salivary flow rate.

Compound (Log P)		Butanoic Acid (0.918)		Ethyl Butanoate (1.492)		2-Heptanone (1.931)		Ethyl Hexanoate (2.405)		3-Octanone (2.598)		2-Nonanone (2.843)	
*m/z*		89		115		117		145		129		143	
		Cmax	RR	Cmax	RR	Cmax	RR	Cmax	RR	Cmax	RR	Cmax	RR
BF	F	15.77		7.09		5.02						26.56	
	*p*-value	0.0005		0.0139		0.0356						<0.0001	
SA	F	23.10		12.51		8.92			9.17	5.22			
	*p*-value	<0.0001		0.0017		0.0064			0.0058	0.0315			
SF	F							13.89	7.24	13.30		6.30	
	*p*-value							<0.0001	0.0035	0.0001		0.0063	
LPM*SA	F						6.04						
	*p*-value						0.0216						
LPM*SF	F				6.11			7.19		6.20			6.95
	*p*-value				0.0213			0.031		0.0201			0.0145
SF*SA	F							5.59	5.55	5.62			5.97
	*p*-value							0.0101	0.0105	0.0099			0.0078

*m/z*: mass/charge ratio; Log P: hydrophobicity of the aroma compounds (calculated with BIOVIA™ discovery studio software (version 2017, BIOVIA™ Corporate Europe, Cambridge, UK) according to Ghose et al. [47]. F: Fisher value calculated by ANOVA; * indicates interaction between parameters.
